# Supplementary material for: Bacterial Evolution in High-Osmolarity Environments
Source: mBio. 2020 Aug 4;11(4):e01191-20. doi: 10.1128/mBio.01191-20 (PMC7407084; doi:10.1128/mBio.01191-20)
Supplement: FIG S1 [file mBio.01191-20-sf001.pdf]

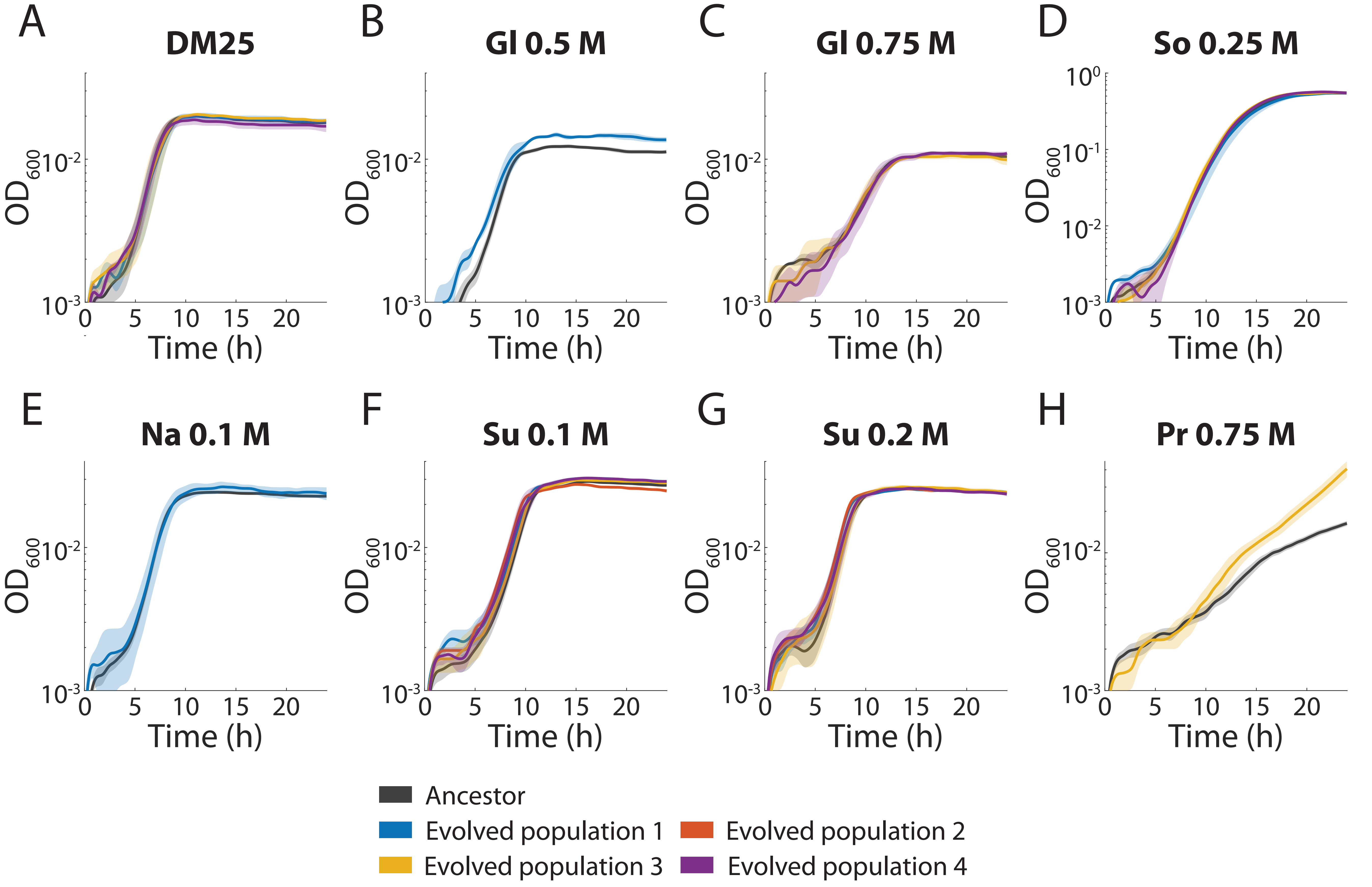

A
DM25
B
Gl 0.5 M
C
Gl 0.75 M
D
So 0.25 M
E
Na 0.1 M
F
Su 0.1 M
G
Su 0.2 M
H
Pr 0.75 M
OD_{600}
10^{0}
10^{-1}
10^{-2}
10^{-3}
0
5
10
15
20
Time (h)
Ancestor
Evolved population 1
Evolved population 2
Evolved population 3
Evolved population 4
