# Supplementary figures and images for: Bacterial Evolution in High-Osmolarity Environments
Source: mBio. 2020 Aug 4;11(4):e01191-20. doi: 10.1128/mBio.01191-20 (PMC7407084; doi:10.1128/mBio.01191-20)

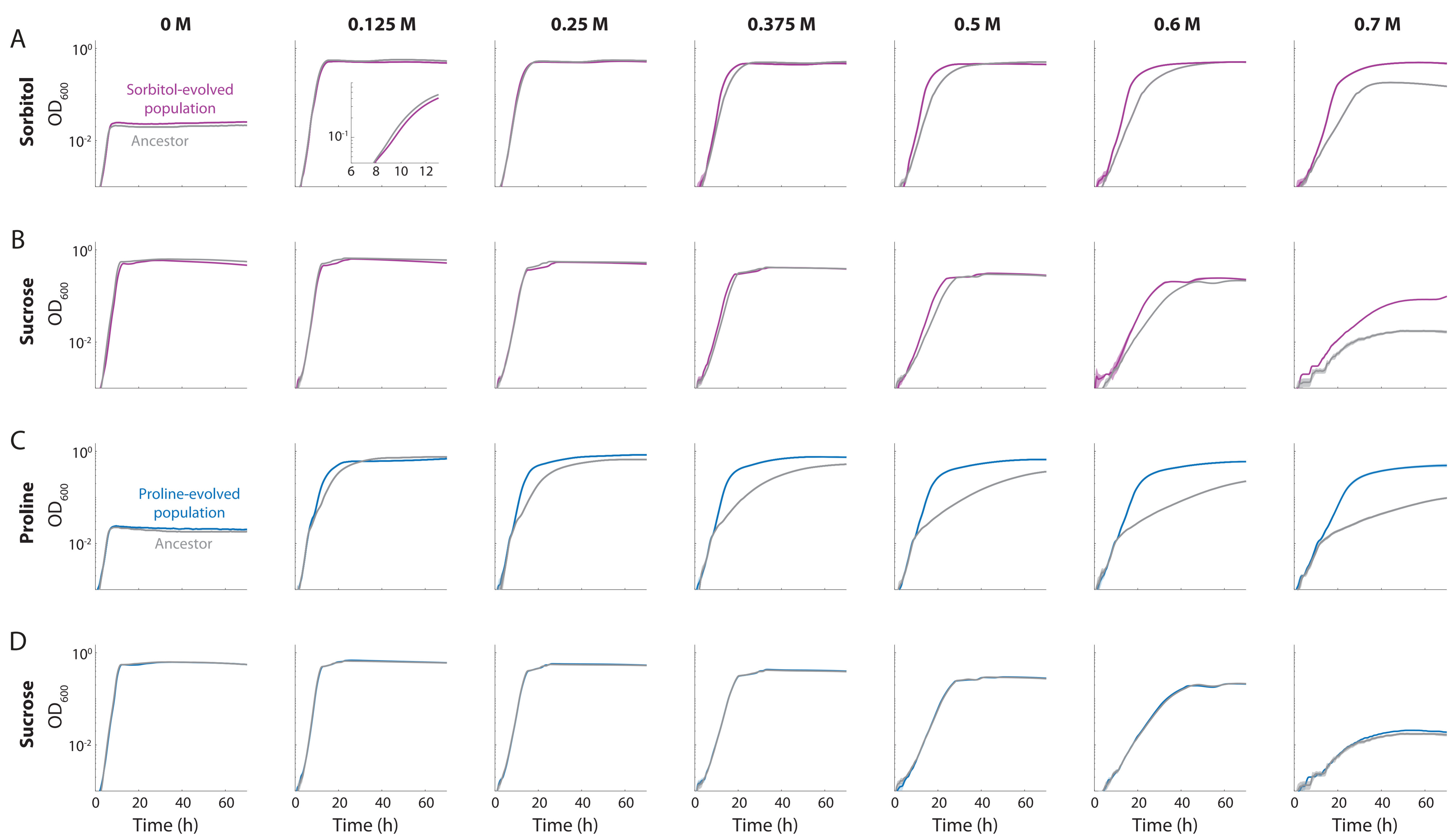

Supplement: FIG S2 [file mBio.01191-20-sf002.pdf]

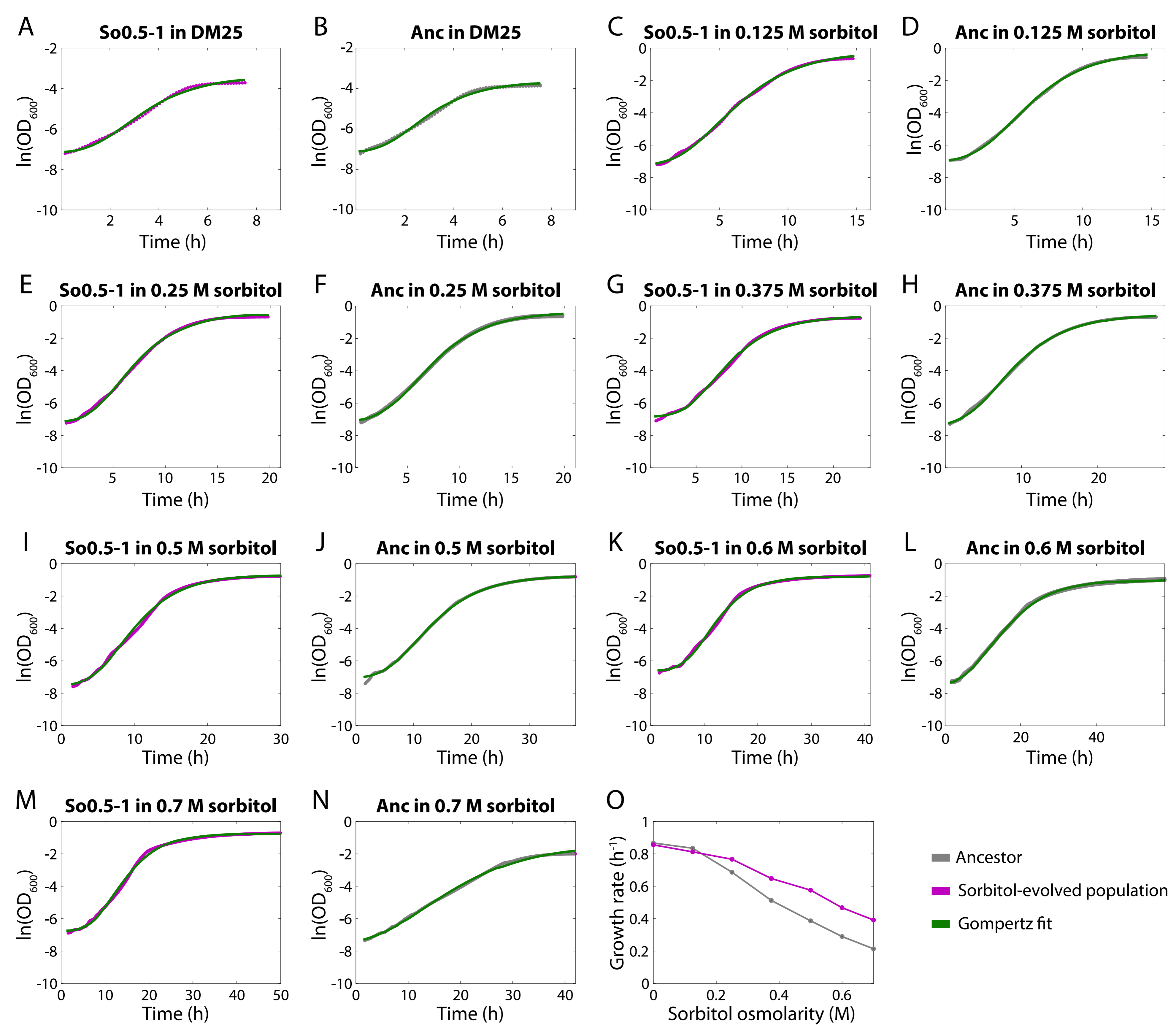

Supplement: FIG S3 [file mBio.01191-20-sf003.pdf]

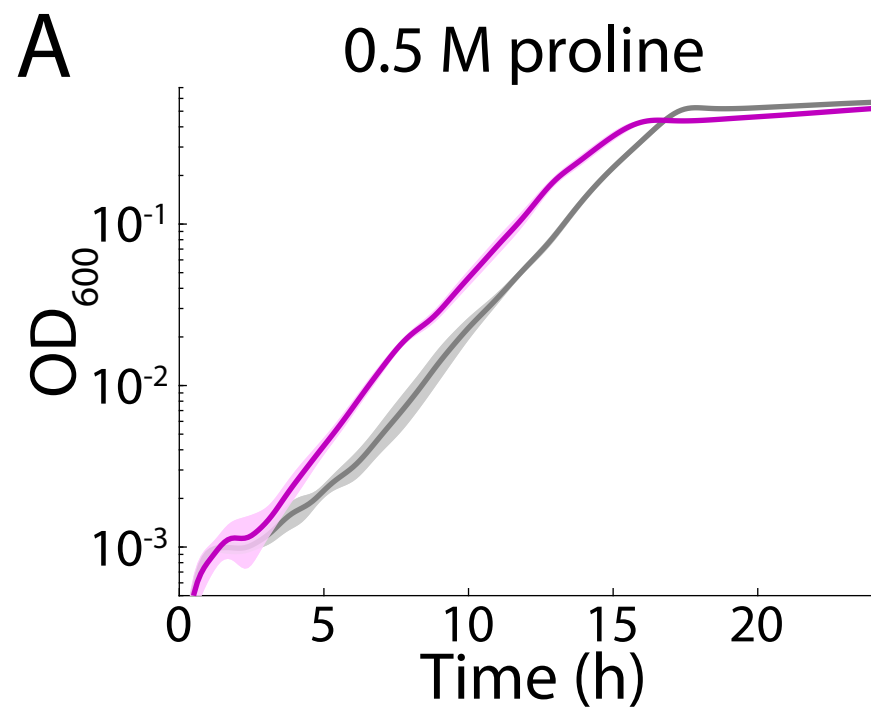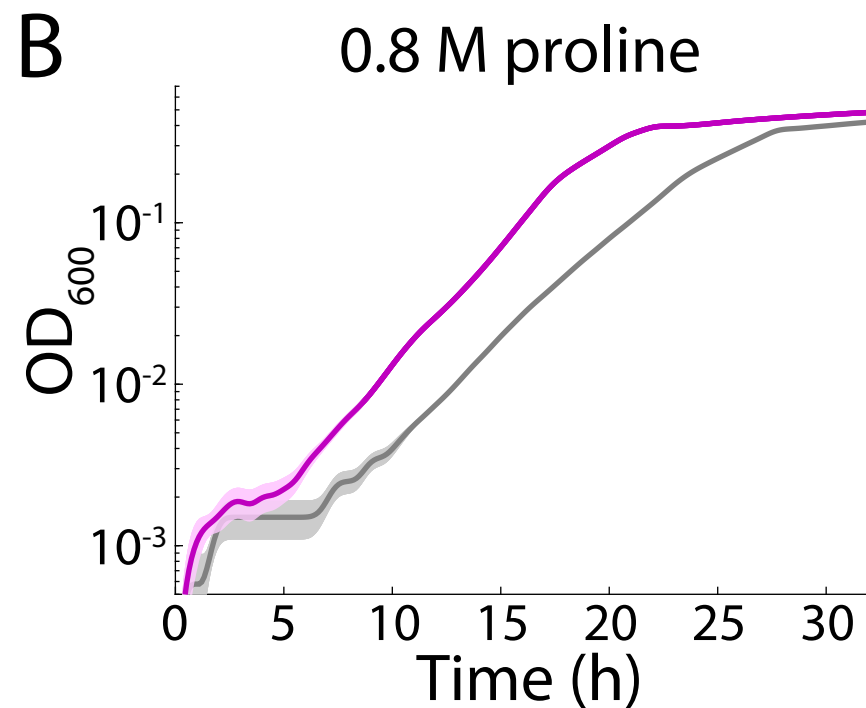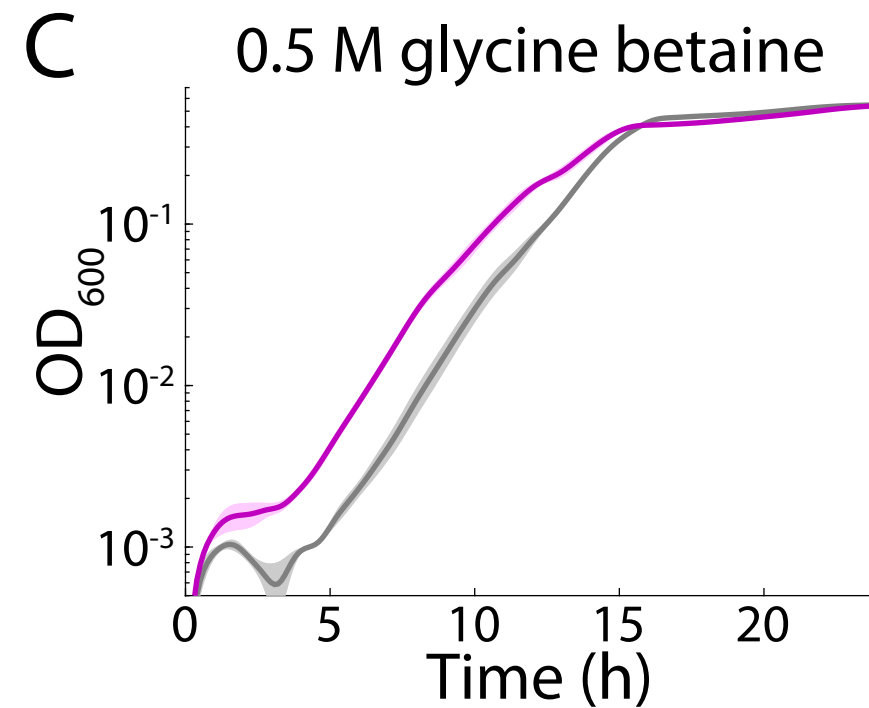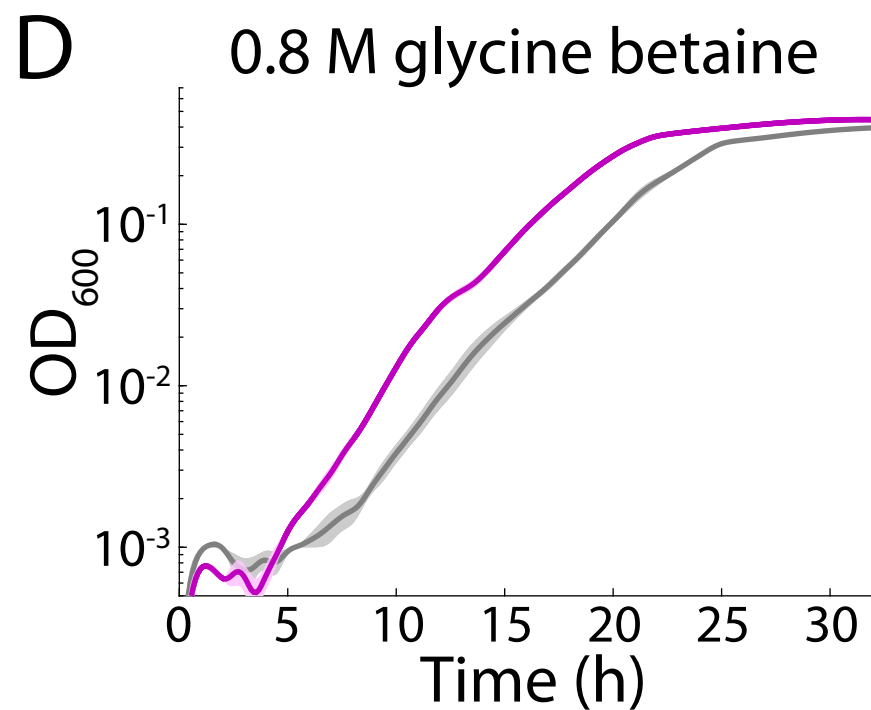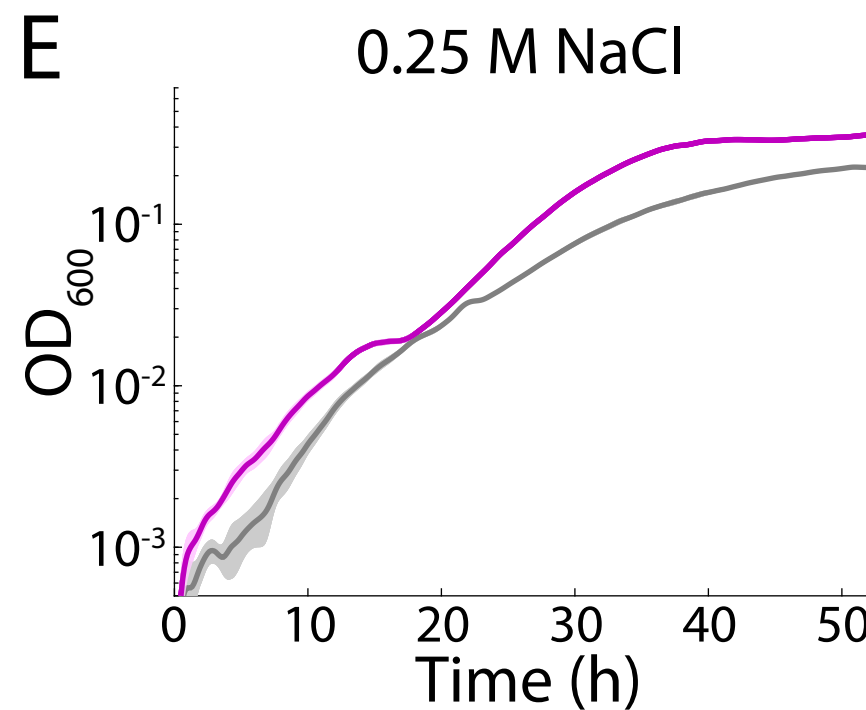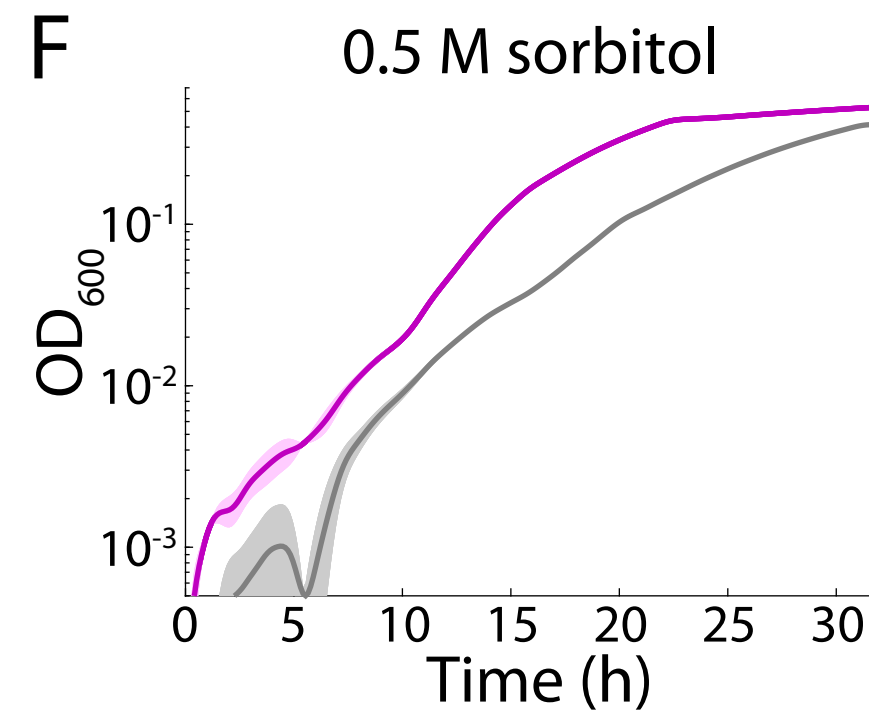

■ Ancestor ■ Sorbitol-evolved population

Supplement: FIG S4 [file mBio.01191-20-sf004.pdf]

**A** 0.4 M sorbitol + 0.1 M proline

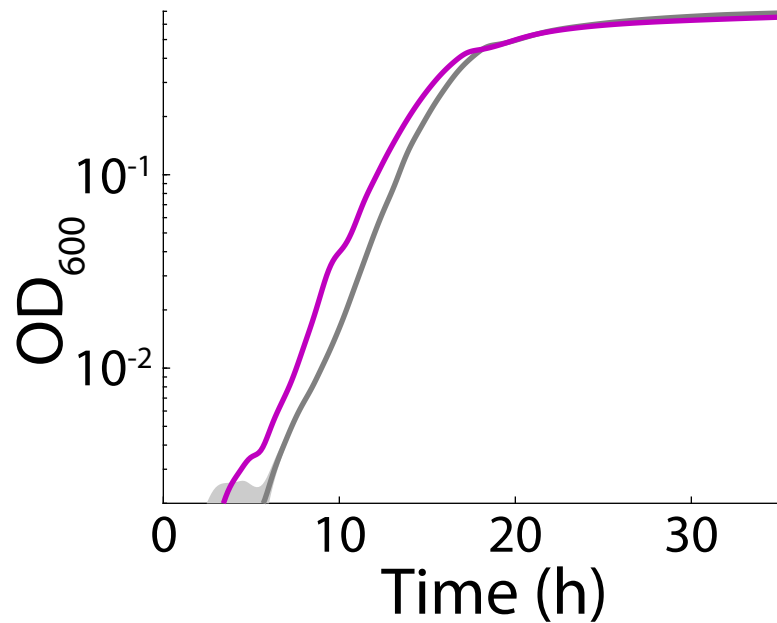

**B** 0.4 M sucrose + 0.1 M proline

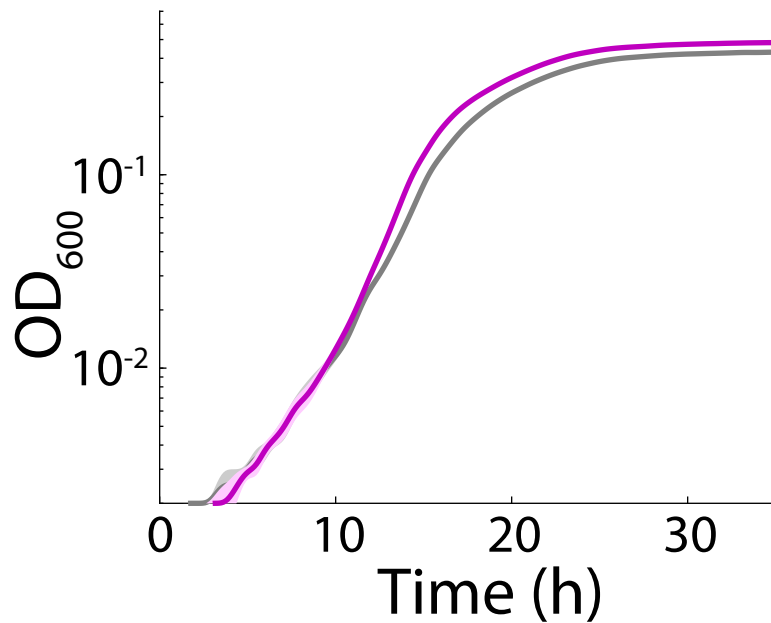

■ Ancestor ■ Sorbitol-evolved population

Supplement: FIG S5 [file mBio.01191-20-sf005.pdf]

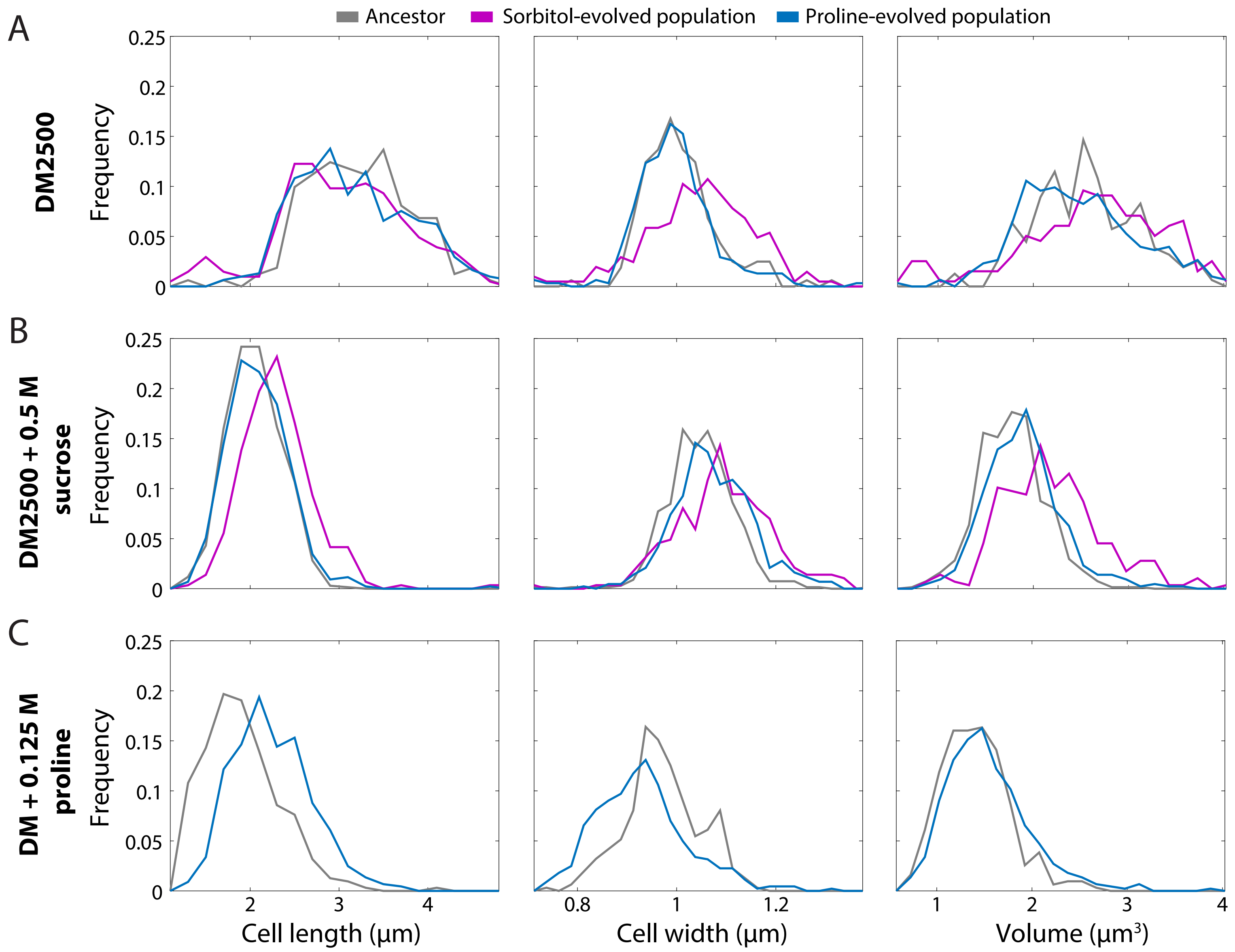

Supplement: FIG S6 [file mBio.01191-20-sf006.pdf]

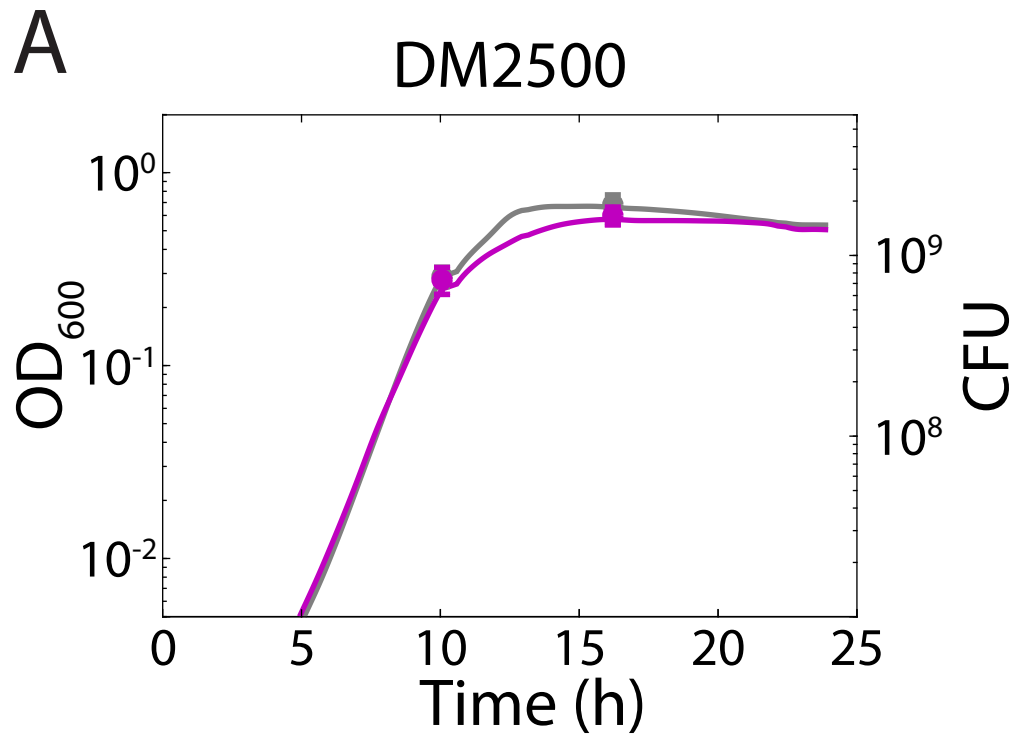

■ Ancestor    ■ Sorbitol-evolved population

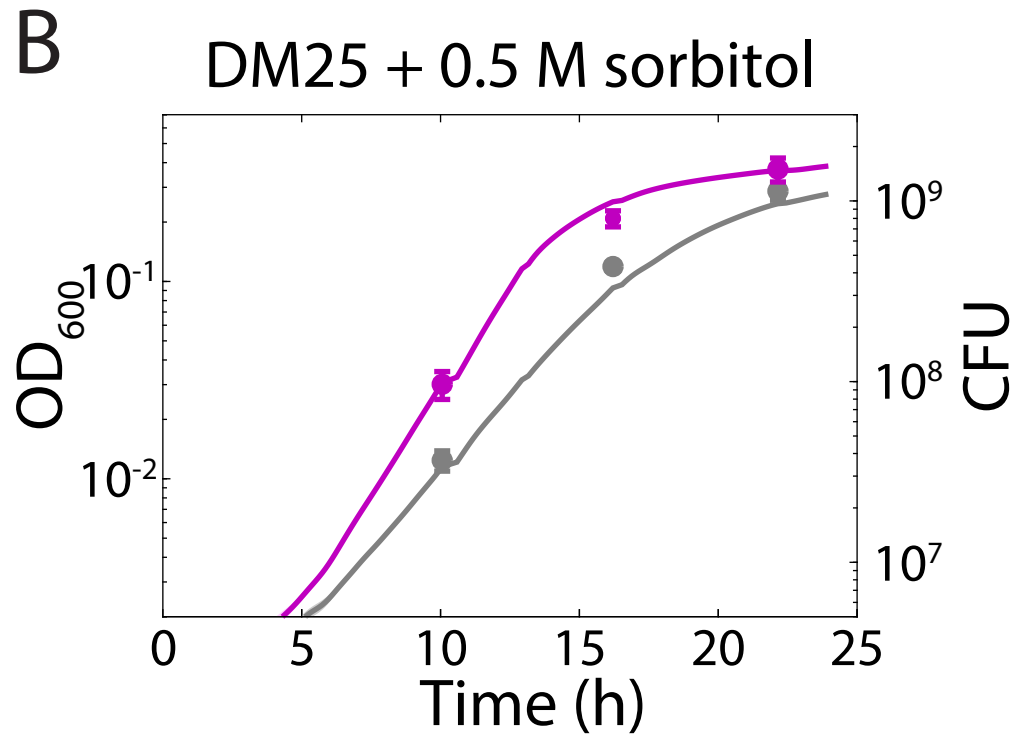

Supplement: FIG S7 [file mBio.01191-20-sf007.pdf]
