# Supplementary material for: Bacterial Evolution in High-Osmolarity Environments
Source: mBio. 2020 Aug 4;11(4):e01191-20. doi: 10.1128/mBio.01191-20 (PMC7407084; doi:10.1128/mBio.01191-20)
Supplement: TABLE S1 [file mBio.01191-20-st001.docx]

**Supplementary Table**

| **Population** | **Position** | **Mutation** | **Frequency** | **Annotation** | **Gene** | **Description** |
| --- | --- | --- | --- | --- | --- | --- |
| So0.5-1 | 1329460 | +CAAAA  CGAT | 0.833 | coding (41/2598 nt) | *topA* → | DNA topoisomerase I |
| So0.5-1 | 1893043 | Δ1 bp | 0.176 | coding (351/2049 nt) | *prc* ← | carboxy-terminal protease for penicillin-binding protein 3 |
| So0.5-1 | 3871975 | IS1 (–)  +8 bp | 0.094 | intergenic (84/+223) | *pstS* ← /  ← *glmS* | phosphate transporter subunit/D-fructose-6-phosphate amidotransferase |
| So0.5-1 | 3928330 | +CTGAAG | 0.091 | coding (114/1260 nt) | *rho* → | transcription termination factor Rho |
| So0.5-2 | 101978 | T→A | 0.184 | F258I (TTT→ATT) | *ftsW* → | integral membrane protein involved in stabilizing FtsZ-ring during cell division |
| So0.5-2 | 386758 | IS1 (–)  +9 bp | 0.614 | coding (447455/  1296 nt) | *phoR* → | sensory histidine kinase in two-component regulatory system with PhoB |
| So0.5-2 | 742545 | Δ132 bp | 0.143 | coding (233364/  1218 nt) | *sucB* → | dihydrolipoamide acetyltransferase |
| So0.5-2 | 3015771 | IS150 (–)  +3 bp | 0.162 | coding (11921194/  1677 nt) | *ECB_02816* → | KpsD protein |
| So0.5-2 | 3251730 | A→G | 0.533 | L344P (CTG→CCG) | *nusA* ← | transcription elongation factor NusA |
| So0.5-2 | 3365992 | G→A | 0.287 | G22S (GGT→AGT) | *mscL* → | large conductance mechanosensitive channel |
| So0.5-2 | 3625458 | T→A | 0.131 | pseudogene (5/  738 nt) | *yhjQ* ← | b3534; cell-division protein (chromosome partitioning ATPase) |
| So0.5-3 | 16972 | IS150 (–)  +3 bp | 0.078 | intergenic (14/514) | *mokC* ← /  → *nhaA* | regulatory protein for HokC, overlaps CDS of *hokC*/pH- dependent sodium/proton antiporter |
| So0.5-3 | 1166458 | T→G | 0.079 | intergenic (+38/50) | *acpP* → /  → *fabF* | acyl carrier protein/3-oxoacyl-(acyl-carrier-protein) synthase |
| So0.5-3 | 1166459 | T→G | 0.069 | intergenic (+39/49) | *acpP* → /  → *fabF* | acyl carrier protein/3-oxoacyl-(acyl-carrier-protein) synthase |
| So0.5-3 | 1434768 | IS150 (+)  +3 bp | 0.107 | coding (112114/  2640 nt) | *ydbH* → | hypothetical protein |
| So0.5-3 | 1462251 | IS150 (–)  +3 bp | 0.275 | intergenic (13/326) | *mokB* ← /  → *trg* | regulatory peptide/methyl-accepting chemotaxis protein III, ribose and galactose sensor receptor |
| So0.5-3 | 1697515 | A→T | 0.094 | Q149L (CAG→CTG) | *slyB* → | outer membrane lipoprotein |
| So0.5-3 | 2416047 | C→A | 0.063 | M119I (ATG→ATT) | *emrY* ← | predicted multidrug efflux system |
| So0.5-3 | 2424024 | IS1 (+)  +9 bp | 0.21 | coding (382390/  945 nt) | *yfdV* ← | predicted transporter |
| So0.5-3 | 2813703 | IS150 (+)  +3 bp | 0.202 | coding (505507/  546 nt) | *syd* ← | SecY interacting protein Syd |
| So0.5-3 | 3968420 | G→T | 0.063 | T65K (ACG→AAG) | *rarD* ← | predicted chloramphenicol resistance permease |
| So0.5-3 | 4096974 | A→C | 1 | F200C (TTT→TGT) | *glpF* ← | glycerol facilitator |
| So0.5-3 | 4504909 | T→C | 0.175 | intergenic (+653/  72) | *insB*25 → /  → *ECB_04162* | b4563(b4576); IS1 protein *insB*/hypothetical protein |
| So0.5-4 | 741343 | A→C | 0.579 | E616A (GAA→GCA) | *sucA* → | alpha-ketoglutarate decarboxylase |
| So0.5-4 | 741844 | +TCA | 0.063 | coding (2348/  2802 nt) | *sucA* → | alpha-ketoglutarate decarboxylase |
| So0.5-4 | 1189282 | IS1 (+)  +9 bp | 0.649 | coding (471479/  1074 nt) | *ycfT* ← | predicted inner membrane protein |
| So0.5-4 | 1331998 | Δ84 bp | 0.25 | coding (2578/2598 nt) | *topA* → | DNA topoisomerase I |
| So0.5-4 | 2647312 | G→A | 0.06 | intergenic (73/  +248) | *kgtP* ← /  ← *rrfG* | alpha-ketoglutarate transporter/5S ribosomal RNA |
| So0.5-4 | 3251631 | Δ27 bp | 0.676 | coding (11041130/  1488 nt) | *nusA* ← | transcription elongation factor NusA |
| So0.5-4 | 3871028 | Δ1 bp | 0.242 | coding (864/  1041 nt) | *pstS* ← | phosphate transporter subunit |
| Pr0.5-1 | 1095148 | C→T | 1 | G7R (GGG→AGG) | *putA* ← | fused DNA-binding transcriptional regulator/proline dehydrogenase/  pyrroline-5-carboxylate dehydrogenase |
| Pr0.5-1 | 3312628 | T→C | 0.101 | L10P (CTA→CCA) | *argR* → | arginine repressor |
| Pr0.5-2 | 858245 | C→A | 0.06 | G224G (GGG→GGT) | *moeA* ← | molybdopterin biosynthesis protein |
| Pr0.5-2 | 877515 | C→A | 0.07 | D90Y (GAC→TAC) | *ybjH* ← | hypothetical protein |
| Pr0.5-2 | 1095079 | G→T | 0.92 | H30N (CAC→AAC) | *putA* ← | fused DNA-binding transcriptional regulator/proline dehydrogenase/  pyrroline-5-carboxylate dehydrogenase |
| Pr0.5-2 | 1592657 | C→A | 0.069 | S40* (TCA→TAA) | *ydeJ* → | competence damage-inducible protein A |
| Pr0.5-2 | 1752551 | C→A | 0.078 | I200I (ATC→ATA) | *aroD* → | 3-dehydroquinate dehydratase |
| Pr0.5-2 | 2451950 | C→A | 0.066 | P88T (CCG→ACG) | *yfeD* → | predicted DNA-binding transcriptional regulator |
| Pr0.5-2 | 3287763 | G→T | 0.147 | D166E (GAC→GAA) | *arcB* ← | hybrid sensory histidine kinase in two-component regulatory system with ArcA |
| Pr0.5-2 | 3625458 | T→A | 0.239 | pseudogene (5/  738 nt) | *yhjQ* ← | b3534; cell-division protein (chromosome partitioning ATPase) |
| Pr0.5-2 | 4586040 | G→T | 0.069 | E273* (GAA→TAA) | *yjjN* → | predicted oxidoreductase, Zn-dependent and NAD(P)binding |
| Pr0.5-3 | 1045926 | T→G | 0.28 | I350L (ATC→CTC) | *yccW* ← | predicted methyltransferase |
| Pr0.5-3 | 1094443 | G→C | 0.692 | P242A (CCG→GCG) | *putA* ← | fused DNA-binding transcriptional regulator/proline dehydrogenase/  pyrroline-5-carboxylate dehydrogenase |
| Pr0.5-3 | 1094878 | C→T | 0.242 | A97T (GCG→ACG) | *putA* ← | fused DNA-binding transcriptional regulator/proline dehydrogenase/  pyrroline-5-carboxylate dehydrogenase |
| Pr0.5-3 | 1095204 | G→A | 0.685 | intergenic (38/385) | *putA* ← /  → *putP* | fused DNA-binding transcriptional regulator/proline dehydrogenase/  pyrroline-5-carboxylate dehydrogenase/  proline:sodium symporter |
| Pr0.5-3 | 2258855 | IS186 (–)  +6 bp | 0.314 | coding (586591/  651 nt) | *alkB* ← | oxidative demethylase of N1-methyladenine or N3-methylcytosine DNA lesions |
| Pr0.5-3 | 3312631 | Δ9 bp | 0.095 | coding (3240/  471 nt) | *argR* → | arginine repressor |
| Pr0.5-3 | 3356398 | T→A | 0.162 | noncoding (270/  1542 nt) | *rrsD* ← | 16S ribosomal RNA |
| Pr0.5-3 | 3463319 | T→G | 0.085 | I412L (ATC→CTC) | *envZ* ← | osmolarity sensor protein |
| Pr0.5-3 | 3602185 | T→G | 0.069 | L118V (TTA→GTA) ‡ | *yhjC* → | predicted DNA-binding transcriptional regulator |
| Pr0.5-3 | 3602186 | T→G | 0.06 | L118* (TTA→TGA) ‡ | *yhjC* → | predicted DNA-binding transcriptional regulator |
| Pr0.5-3 | 3625458 | T→A | 0.121 | pseudogene (5/  738 nt) | *yhjQ* ← | b3534; cell division protein (chromosome partitioning ATPase) |
| Pr0.5-3 | 4141540 | IS1 (+)  +8 bp | 0.084 | coding (613620/  705 nt) | *yijC* → | DNA-binding transcriptional repressor |
| Pr0.5-4 | 1094872 | A→C | 1 | Y99D (TAT→GAT) | *putA* ← | fused DNA-binding transcriptional regulator/proline dehydrogenase/  pyrroline5carboxylate dehydrogenase |
| Pr0.5-4 | 1322850 | C→A | 1 | A25A (GCC→GCA) | *insA*11 → | IS1 protein InsA |
| Pr0.5-4 | 3127587 | G→T | 0.082 | intergenic (+226/  +567) | *yqiK* → /  ← *rfaE* | hypothetical protein/fused heptose 7-phosphate kinase/heptose 1-phosphate adenyltransferase |
| Pr0.5-4 | 4628175 | C→T | 0.348 | D98N (GAT→AAT) | *arcA* ← | DNA-binding response regulator in two-component regulatory system with ArcB or CpxA |
| Pr0.5-4 | 4358156 | +A | 0.137 | coding (328/396 nt) | *frdC* ← | fumarate reductase subunit C |

**Table S1: Mutations identified via metagenomic sequencing of the So0.5 and Pr0.5 populations.** Mutations that were present at <5% were not included in the table.
